# The Genus Hyssopus: Traditional Use, Phytochemicals and Pharmacological Properties

**DOI:** 10.3390/plants13121683

**Published:** 2024-06-18

**Authors:** Gayane Atazhanova, Margarita Ishmuratova, Yana Levaya, Marlen Smagulov, Yekaterina Lakomkina

**Affiliations:** 1 Research Park of Biotechnology and Eco-Monitoring, Karaganda Buketov University, Universitetskaya Street, 28, Karaganda 100028, Kazakhstan; g-atazhanova@mail.ru (G.A.); marlenkemel@mail.ru (M.S.); 2 School of Pharmacy, Karaganda Medical University, Gogol Street, 40, Karaganda 100017, Kazakhstan; ekaterina.yankovskaya@inbox.ru

**Keywords:** *Hyssopus*, distribution, compounds, essential oils, flavonoids, terpenoids, biological activity

## Abstract

According to modern concepts, the genus *Hyssopus* L. includes seven plant species (*Hyssopus ambiguus* (Trautv.) Iljin ex Prochorov. & Lebel; *Hyssopus cuspidatus* Boriss; *Hyssopus latilabiatus* C.Y.Wu & H.W. Li; *Hyssopus macranthus* Boriss.; *Hyssopus officinalis* L.; *Hyssopus seravschanicus* (Dubj.) Pazij; *Hyssopus subulifolius* (Rech.f.) Rech.f.). The plants are rich in various groups of biologically active substances with a wide spectrum of pharmacological action. This review presents a modern comprehensive overview of the botanical research, extraction methods, chemical composition and pharmacological activity of plants of the genus *Hyssopus* L. As a result of the review, it was established that the chemical composition of plant extracts of the genus *Hyssopus* L. depends on various factors (place of growth, weather conditions, chemotypes, extraction methods, etc.). For the further use of the plants, the extraction methods and low-molecular metabolites isolated from them (mono- and sesquiterpenoids, flavonoids, alkaloids, etc.) are discussed. The data from the review provide an assessment of the relevance.

## 1. Introduction

Currently, the genus *Hyssopus* L. (family Lamiaceae or Labiatae) has seven species [1,2], being one of the small genera. According to the Plants of the World Online (POWO) online taxonomic list [3,4], the genus *Hyssopus* is confirmed as including seven species distributed naturally in Europe, Asia, northern Africa, and also introduced in North America. These comprise *Hyssopus ambiguus* (Trautv.) Iljin ex Prochorov. & Lebel, *Hyssopus cuspidatus* Boriss., *Hyssopus latilabiatus* C.Y.Wu & H.W. Li, *Hyssopus macranthus* Boriss., *Hyssopus officinalis* L., *Hyssopus seravschanicus* (Dubj.) Pazij and *Hyssopus subulifolius* (Rech.f.) Rech.f. Moreover, according to World Flora Online (WFO) [4], *H. ferganensis* Boriss. and *H. tianschanicus* Boriss. are defined as a synonym for *H. seravschanicus*.

In the flora of Kazakhstan [5], the following four species are described: *H. cuspidatus* Boriss., *H. ambiguus* Iljin, *H. macranthus* Boriss. and *H. tianschanicus* Boriss. Later, M.S. Baitenov [6] listed approximately 15 species distributed in Eurasia from the Mediterranean Sea to Central Asia, confirming the presence of only four species in the territory of Kazakhstan.

*Hyssopus* has been known as a medicinal plant since the time of Hippocrates (circa 460–377 BC), who mentioned it in his writings. The most common representative of the genus is *H. officinalis* L. This species (leaves and flowers) is widely used in traditional medicine, cooking and perfumery. *H. officinalis* is widely found in Europe and North Africa. This plant is included in the official pharmacopoeias of France, Portugal, Romania, Sweden and Germany [7]; the herb is actively used in the food industry [8].

In the genus *Hyssopus*, the plants’ main function is providing essential oil for the cosmetic and perfume industry, especially for the production of oriental fragrances [9]. Hyssop essential oil exhibits antibacterial, antiviral and expectorant properties. This makes it an important ingredient in aromatherapy, pharmaceuticals, personal care products, food and beverages [9].

However, in our time, the culture of hyssop has been unnecessarily overlooked, with its industrial use significantly limited. The plants of the genus *Hyssopus* L. have potential for further research on the development of medicinal and cosmetic products with relatively high biological properties.

For a comprehensive review of the literature, we analyzed published data available through the following search engines: SciFinder^®^, Web of Science^®^, Scopus^®^ and Google Scholar^®^. When searching for information, the following keywords were used: “Hyssopus”, “compounds”, “isolation”, “extraction” and “activity”. The available reviews either presented the phytochemical and pharmacological properties of only one species, *H. officinalis* L., or the reviews were devoted to one class of chemical compounds [8,9,10,11]; meanwhile, there were no reviews about other plants’ properties in the species *Hyssopus* L. In this regard, this paper aims to elucidate, using new data, the chemical composition of plants of the genus *H. officinalis* L., the pharmacological properties of isolated extracts, essential oils and individual compounds and their further use in the pharmaceutical, cosmetic and medical industries.

Plants of the genus Hyssop, like many other plants, contain various classes of biologically active compounds, such as flavonoids, essential oils, phenolic compounds and others. The purpose of our review is to collect and systematize the available data on the chemical composition of these plants and their potential pharmacological properties, since the Republic of Kazakhstan is actively developing the technology of new plant-based medicines, and possesses significant reserves of some species of Hyssop, such as *H. cuspidatus* Boriss., *H. ambiguus* Iljin, *H. macranthus* Boriss. and *H. tianschanicus* Boriss. In many countries, plants of the genus Hyssopus are traditionally used for medicinal, perfumery, cosmetic and nutritional purposes. Extensive research into the content of biologically active compounds in *Hyssopus macranthus* Boriss. and *Hyssopus subulifolius* (Rech.f.) has not yet been conducted, and in relation to *H. angustifolius* M. Bieb., *H. tianschanicus* Boriss., *H. ambiguus* Iljin, *H. cretaceus Dub.*, *H. seravschanicus* (Dub.) Pazij and *H. ferganensis* Boriss, only individual classes have been studied to date. This article will help evaluate the scientific evidence for hyssop’s traditional use and determine its effectiveness and safety. In addition, further research into the chemical composition of hyssop plants may lead to the isolation of new biologically active compounds that can be used to create new medicines for clinical use.

## 2. Botany

The plants of this genus are characterized by the presence of elongated or oblong inflorescences, multi-flowered, spike-shaped and consisting of close or spread whorls, sitting in the axils of the leaves. The calyx is tubular-bell-shaped, with 15 veins, almost regular, with five almost equal teeth. The calyx is covered with glands, its surface is painted lilac-green and the inside is bare. The corolla is fused-petalled, two-lipped, hairy and with glands. The upper lip is almost flat, notched or bilobed; the lower one is three-lobed, with a larger middle blade. There are four stamens, one style, bifid at the apex and the nuts are oblong or oblong–ovate. The leaves range from linear to oblong. Life forms are represented by perennial herbaceous plants or subshrubs. The general distribution of representatives of the genus covers Europe, Asia and North Africa; additionally, introduced species have been noted in North America (Table 1). 

Thus, the main differences between the species are the structure of the leaf shape, the size of the inflorescences and the structure of the calyx and corolla of the flower. The habitat of all species is confined mainly to arid territories (mountain slopes, steppes), rocky or sandy soils. In the territory of Kazakhstan, three species have a wide range beyond its borders, covering Western Siberia, Mongolia, Central Asia and the Tien Shan. One species is endemic, whose range includes the northern, central and eastern territories [5]. Not all species are sufficiently studied botanically and chemically. 

## 3. Methods for Isolating Extracts and Essential Oils from Plants of the Genus *Hyssopus* L.

Various methods and solvents are used to isolate polar and nonpolar secondary metabolites from plants of the genus *Hyssopus*. To isolate the components of essential oils, steam distillation with hydro-distillation is traditionally performed using either a Clevenger-type apparatus and the aerial parts of plants with a distillation time of 2–3 h [11] or a Dean–Stark apparatus with a distillation time of up to 4 h [12]. The volatile components from *H. officinalis* were isolated using the Soxhlet extraction method using pentane/diethyl ether and supercritical extraction with carbon dioxide [13]. The use of supercritical fluid extraction for *Hyssopus* at different conditions, including pressure, temperature, extraction times and modifier concentrations, using an orthogonal lattice design with matrix conditions influenced the extraction yield of major monoterpenoids [14]. In [15], the supercritical extraction of *H. officinalis* was carried out using carbon dioxide as an extractant. The effect of pressure (80, 100 and 150 bar) on the yield of the total extract was studied at a temperature of 313 K, a flow rate of 0.00323 kg/min and an average particle diameter of 0.49 mm, obtaining a strong correlation between the inverse values of the total extract yield and extraction time.

However, these methods also have disadvantages: they were developed only in laboratory conditions or in pilot plants, and require expensive and bulky equipment. To isolate the essential oil from *H. officinalis*, an effective and economically attractive technology, Détente Instantanée Contrôlée (DIC), was proposed. This is a thermomechanical process that involves exposing the raw material to saturated steam under high pressure for a brief duration, followed with a sharp drop in pressure inside the vacuum. In relation to other methods, DIC has several other benefits, including no solvents used, a higher extract quality making it environmentally friendly on an industrial scale, with a high speed, selectivity, automatic operation and performance under normal conditions. At the same time, the yield of *H. officinalis* extract turned out to be the highest when compared to the methods of hydro-distillation, ultrasonic extraction and the Soxhlet method [16]. For a relatively high yield of essential oil, the plant should be collected at the full-flowering stage.

Many methods have been used to extract and isolate plant phytochemicals from *H. officinalis*, such as homogenization, solvent extraction, maceration, grinding, ultrasonication and Soxhlet extraction.

The authors of [17] proposed an innovative method for obtaining essential oil, which included the following stages: preliminary grinding, mixing with a reagent, infusion at temperature and the ratio of material and reagent, and hydro-distillation to obtain essential oil. The grinding of herbal essential oil raw materials was carried out to sizes of 5–15 mm, mixing with the reagent in a volume ratio from 1:5 to 1:8 and infusing the raw materials at a temperature of 22 to 24 °C for 3 to 5 h. Electro-activated water with a pH of 8.0 to 9.5 was used as a reagent, obtained via electrolysis of a 1–2% aqueous solution of NaCl, at a current of 0.5–0.6 A and a voltage of 36 V. The yield of hyssop essential oil’s medicinal content using the proposed technology ranged from 0.6 to 0.8%. The quality of the essential oil of *H. officinalis* was assessed using the ratio of the main components—pinocamphone and cis-pinocamphone—to the total content of essential oil components [17].

Ultrasonic extraction is one of the modern methods for obtaining compounds from plant organs. In [18], the authors obtained *H. officinalis* leaf extract using ultrasonic extraction with an ethanol/water/solvent ratio (50:50) and (80:20) from 10 to 20 min at 30 and 40 °C. The (80-40-20) solution identified the highest amount of antioxidant activity in the inhibition of DPPH radicals and beta-carotene–linoleic acid color analysis and determined the highest amount for phenolic compounds (193.3 ± 5.53 mg/g) and flavonoids (40.63 ± 2.36 mg/g).

A relatively high content of extractives was observed during the microwave extraction of *H. officinalis* (g/100 g of dry weight)—23.4 ± 0.36; with ultrasonic cavitation—20.6 ± 0.48; during extraction in the Soxhlet apparatus—15.1 ± 0.25 and during maceration—12.4 ± 0.14. However, the extraction methods also influenced the concentration of phenolic compounds in *H. officinalis*, with the highest to the lowest percentage of phenolic compounds as follows: microwave extraction > ultrasonic extraction > Soxhlet extraction > maceration [19].

Ahmadian et al. [20] demonstrated that ultrasound combined with cold atmospheric plasma as a pre-treatment improved the extraction of phenolic components from *H. officinalis* by approximately 22% compared to the use of ultrasound alone.

To obtain various extracts, polar (acetone, methanol, ethanol) and nonpolar solvents (hexane, petroleum ether) were used. The polar solvents were used more frequently and provided better results, both in terms of the concentration and biological potential. For a relatively high yield of extractives, the best solvent was aqueous alcohol at 70% [21].

To obtain extractives from the structure of the material, an extraction process using new physical methods can be used. One of the promising methods for intensifying extraction is the electrophysical method, where the material is treated with a pulsed electric field, which can be applied to substances that are polar dielectrics in physical nature. The authors of [22] presented the results of the intensification processes of polysaccharides from *H. officinalis* under the influence of electric current. The energy consumption for the *H. officinalis* extraction process was proved to be intensified with a pulsed electric current that was significantly lower than extraction via convection heating. The possibility of increasing the content of extractable polysaccharides by 48% after extraction was demonstrated. That is, this process makes it possible to reduce by three times the time required for obtaining water-soluble polysaccharides compared to traditional pharmacopoeia convection methods and, furthermore, to reduce energy costs by 20 times. The use of electric current can also lead to a reduction in the maximum processing temperature to 40 °C, which makes it possible to obtain aqueous alcoholic and alcoholic extracts, and to extract biologically active substances that are insoluble in water.

In summary, a literature review revealed a large number of extraction methods from the plants of the genus *Hyssopus*. Each of the traditional methods has its own advantages and disadvantages; the choice of one or another option depends on the purpose of using the processor. Therefore, the choice of the extraction system should be based on a careful analysis of the essential properties of the extract and its components.

## 4. Mono- and Sesquiterpenoids of Essential Oils from Plants of the Genus *Hyssopus* L.

The essential oils from plants of the genus *Hyssopus* are known for their medicinal and aromatic properties. These oils have antimicrobial, antiviral and expectorant properties, making them a valuable ingredient in aromatherapy, pharmaceuticals, personal care products, food and beverages. The most common *H. officinalis* essential oil is produced and distributed by various companies, including Now Foods, Katyani Exports, Ungerer & Company, Young Living, doTERRA, Edens Garden, Radha Beauty, Majestic Pure, Art Naturals, Healing Solutions, Native American Nutritionals and Rocky Mountain Oils.

The *H. officinalis* essential oil market has experienced significant growth in recent years, stimulated by the increased consumer attention toward the benefits of natural and organic products, the growing demand for alternative medicine and rising incomes. The market research shows that the *H. officinalis* essential oil market is poised for sustained growth, with opportunities for manufacturers, suppliers and distributors to capitalize on the rising demand. In connection with economic use, a more thorough study of the chemical composition of the *H. officinalis* essential oil and other species of this genus is necessary [23]. The herb *H. officinalis* is included as an official raw material in the pharmacopoeias of France, Portugal, Romania, Sweden and Germany.

An analysis of the available literature devoted to studying the composition of the *H. officinalis* essential oil showed that the information is fragmentary and often contradictory. Most frequently, summary data are provided on the quantitative content of the dominant components; in some cases, there is an analysis of the component composition of various morphological forms.

The component composition of the *H. officinalis* essential oil, which grows in various geographical areas, is reasonably well known. For example, studies of the chemical composition of the ethereal *H. officinalis* of various chemotypes (pinocamphonic, linalool, thymolic) are described. The data on the composition of the *H. ambiguus* (Trautv.) Iljin ex Prochorov. & Lebel, *H. cuspidatus* Boriss., *H. officinalis* L. and *H. seavschanicus* (Dubj.) Pazij essential oils are presented in Table 2.

**Figure 1:**
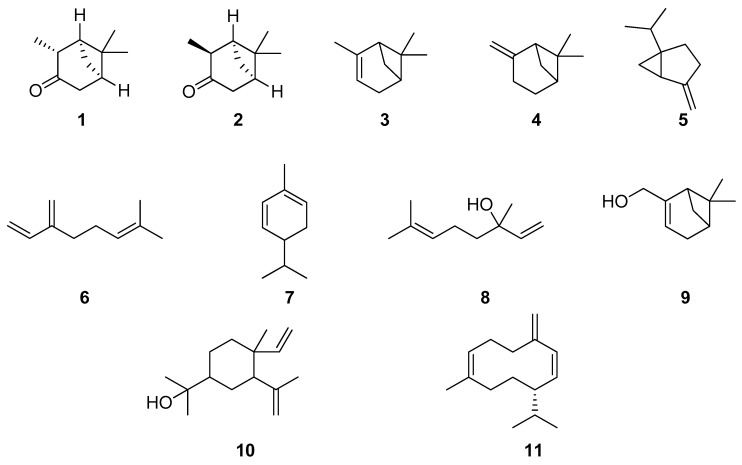
Chemical structures of mono- and sesquiterpene molecules from plants of the genus Hyssop.

Many publications are devoted to the study of the species *H. officinalis* L. growing in European, Asian and African countries. The component composition and quantitative content of various constituents in essential oils may vary depending on soil, climatic and genetic factors [12,30,31,32,33,34,35,36,37,38,39,40,41,42,43,44,45,46,47,48,49,50,51,52,53,54,55,56,57,58,59,60,61,62]; however, the main ketones that are characteristic of this species are pinocamphone **1** and cis-pinocamphone **2** (their relative content varies ranging from 2.94 to 63.55%; these components are in dynamic equilibrium), α-pinene **3** and β-pinene **4**, sabinene **5**, myrcene **6**, phellandrene **7**, linalool **8**, myrtenol **9**, elemol **10** and germacrene-D **11** (Figure 1).

Blue-flowered plants reportedly contain more essential oil than pink- and white-flowered forms. In addition, plants with different flower colors have differences in the percentage of specific essential oil components [67]. Chromato-mass-spectral analysis of the content of volatile organic compounds in plants of the same variety, but differing in flower color, revealed features in the biosynthesis of secondary metabolites. The studies have shown that, in the white-flowered plants, the content of pinocamphone **1** was up to 44.99%, in the blue-flowered plants it was up to 20.85% and in the pink-flowered plants it was up to 45.23% [37].

In [68], details are provided of a study conducted to examine the component composition of the essential oil of forms of *H. officinalis*, manifested in white, blue and pink flowers. The study revealed that there are no significant differences in the hydrocarbon content between the white- and pink-flowered forms. However, the blue-flowered form had half the hydrocarbon content (4.4%). The white-flowered form had a high alcohol content (up to 8.69%), while the blue-flowered (up to 5.73%) and pink-flowered (up to 4.61%) forms had a lower alcohol content. In the blue- and pink-flowered forms, the content of aldehydes and ketones was the same (59.8% each); meanwhile, in the white-flowered form, it was slightly higher (up to 62.17%).

According to the studies, most species of *H. officinalis* synthesized components such as pinocamphone **1**, cis-pinocamphone **2**, β-pinene **4**, sabinene **5**, myrtenol **9** and elemol **10**, as well as some others in small quantities. The second group of plants (20%) concentrated five main mono- and sesquiterpenoids, mainly consisting of pinocamphone **1** (up to 60%), β-pinene **4** (up to 6.2%), β-phellandrene **7** (up to 6.8%), spathulenol **12** (up to 3.5%) and myrtenol **9** (up to 6.3%), as well as (Z)-caryophyllene **13** (up to 3.5%). The third group of plants (10%), including *H. officinalis*, differed in their synthesis, mainly producing cis-pinocamphone **2** (up to 61.1%), β-pinene **4** (up to 10.5%), elemol **10** (up to 19%), β-eudesmol **14** (up to 7.6%) and a small amount of sesquiterpenes (up to 25%). The essential oil of all studied plants corresponded to the composition of *H. officinalis*, differing in the quantitative content of the main components. The content of individual hydrocarbons in the essential oil did not exceed 1.5%, and the largest amount of β-pinene **4** was 10.5%.

**Figure 2:**
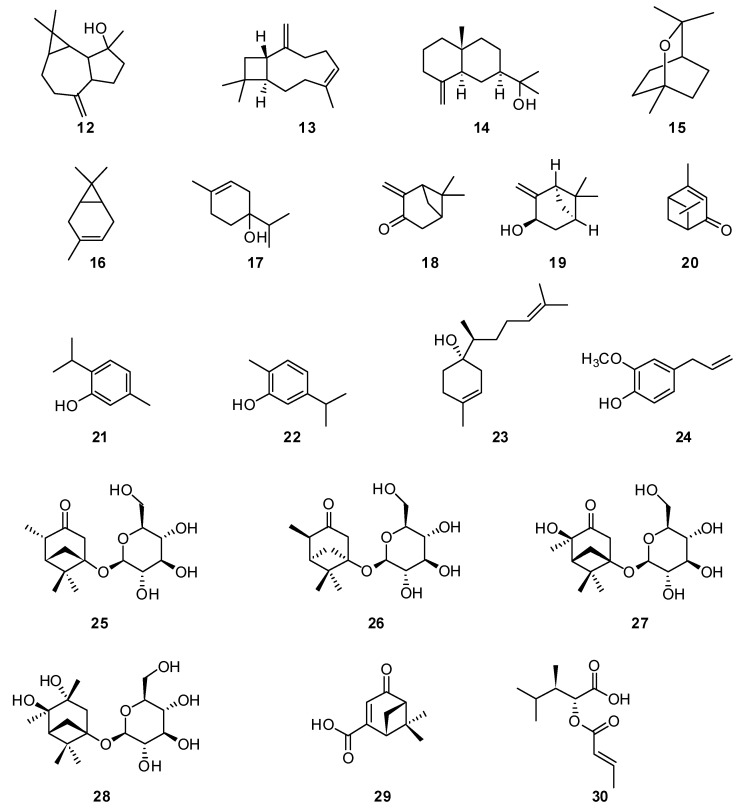
Chemical structures of isolated mono- and sesquiterpenes from plants of the genus Hyssop.

The main component of the *H. ambiguus* [24,25] and *H. cuspidatus* essential oils [26] is 1,8-cineole **15** (Figure 2). Samples of *H. ambiguus* essential oil were collected in the vicinity of the town of Karkaralinsk and the neighboring village. The varieties differ in qualitative and quantitative composition. Thus, in samples from the first point of growth, 3-carene **16**, terpinen-4-ol **17** and germacrene D **11** were identified; meanwhile, in the raw materials from the second collection point, β-pinene **4** and β-myrcene **6** were identified. In Spain, 1,8-cineole **15** is a major component of the *H. officinalis* essential oil [61]. β-pinene **4**, 1,8-cineole **15** and cis-pinocamphone **2** are the main compounds found in wild plants of the *H. officinalis* subspecies from Serbia [69], which also formed a major component in *H. officinalis* essential oil from Bulgaria [55].

Moreover, 1,8-cineole **15** was also found in samples of *H. cuspidatus* essential oils growing in Altai and China [26,27]. The main components in these oils are pinocarvone **18** (27.06%), 1,8-cineole **15** (10.76%) and cis-pinocarveol **19** (9.57%). In Altai, the components were verbenone **20** (23.84%), β-pinene **4** (19.76%), pinocamphone **1** (17.95%), 1,8-cineole **15** (7.16%) and myrtenol **9** (7.06%). Thymol **21** was the main component of *H. cuspidatus* [29], growing in Taicheng, Xinjiang, China. In addition, thymol **21** (18.95%) was found in the *H. officinalis* essential oil of from Iran, and carvacrol **22** (7.73%) and β-bisabolol **23** (16.62%) were also found [70].

The monoterpenoid linalool **8** was found in significant quantities in the *H. officinalis* essential oil from France, amounting to almost 50%. In [71], 44 chemical constituents were detected in the *H. officinalis* essential oil cultivated in Italy using GC–MS analysis. The main chemical constituents detected were linalool **8** (47.7%) and methyl eugenol **24** (9.9%) [71].

In addition to the known compounds, six previously undescribed monoterpenoids **25**–**30** were isolated and identified from the n-BuOH fraction of *H. cuspidatus* [72]: 

**25**—(1S,4S,5S)-4,6,6-trimethyl-1-(((2R,3S,4R,5R,6S)-3,4,5-trihydroxy-6-(hydroxymethyl)tetrahydro-2H-pyran-2-yl)oxy)bicyclo[3.1.1]heptan-3-one.

**26**—(1R,4R,5R)-4,6,6-trimethyl-1-(((2R,3S,4R,5R,6S)-3,4,5-trihydroxy-6-(hydroxymethyl)tetrahydro-2H-pyran-2-yl)oxy)bicyclo[3.1.1]heptan-3-one.

**27**—(1S,4R,5R)-4-hydroxy-4,6,6-trimethyl-1-(((2R,3S,4R,5R,6S)-3,4,5-trihydroxy-6-(hydroxymethyl)tetrahydro-2H-pyran-2-yl)oxy)bicyclo[3.1.1]heptan-3-one.

**28**—(2R,3S,4R,5R,6S)-2-(((1S,3R,4R,5R)-3,4-dihydroxy-3,4,6,6-tetramethylbicyclo[3.1.1]heptan-1-yl)oxy)-6-(hydroxymethyl)tetrahydro-2H-pyran-3,4,5-triol.

**29**—(1R,5S)-6,6-dimethyl-4-oxobicyclo[3.1.1]hept-2-ene-2-carboxylic acid.

**30**—(2R,3R)-2-((E)-but-2-enoyloxy)-3,4-dimethylpentanoic acid.

Thus, a significant amount of information has been identified in the literature, studying the component composition of essential oils from plants of the genus *Hyssopus*. The genus *Hyssopus* is characterized by the representation of mono- and sesquiterpenoids of all biogenetic lines; in particular, the pinocamphone **1** line is most developed in *H. officinalis*, where it comprises more than half of the essential oil, reaching a maximum of 90%. The biogenetic lineage of cis-pinocamphone **2** is more common and was identified as the main component in almost a third of the species considered. In some species of *Hyssopus*, the essential oils contain large amounts of linalool **8**, thymol **17** and 1,8-cineole **15**, which are found in other species of the family *Lamiaceae*.

## 5. Steroids and Triterpenoids of Plants of the Genus *Hyssopus* L.

**Figure 3:**
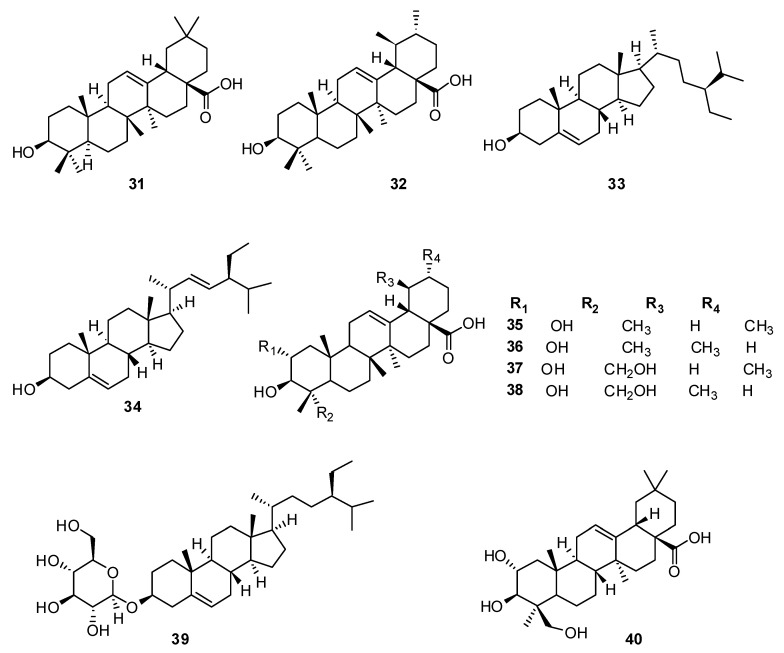
Chemical structures of triterpenoid and steroid molecules from plants of the genus Hyssop.

Many species of the family Lamiaceae accumulate significant amounts of triterpenoids, which are structurally and genetically similar to steroids. Of particular interest are the pentacyclic triterpene acids—ursolic **31** and oleanolic **32**—which were found in the raw materials of some *Hyssopus* species (Figure 3) [73].

The experiments showed that chloroform and 70% alcohol extracts obtained from the herb *H. officinalis* contain oleanolic acid **32** and ursolic acid **31**. The best separation of triterpenoids occurred in the system petroleum ether–chloroform–acetic acid (10:4:0.4) [74].

Oleanolic acid **32**, ursolic acid **31** and β-sitosterol **33** were isolated from the ethyl acetate fraction of the *H. seravshanicus* herb using CC and Sephadex LH-20 column chromatography in combination with semipreparative HPLC [75].

The authors of [76] studied the cell cultures of *H. officinalis* as a means of learning the extent of their ability to synthesize secondary metabolites. The TLC analysis of dichloromethane extracts of cultured cells revealed the presence of sterols and triterpenes [76].

Cell suspension cultures from *H. officinalis* hypocotyl-derived callus were found to produce two sterols named β-sitosterol **33** and stigmasterol **34**; additionally, a number of known pentacyclic triterpenes with an oleanane and ursine skeleton were found. The triterpenes were recognized as oleanolic acid **32**, ursolic acid **31**, 2α, 3β-dihydroxyolean-12-en-28-oic acid **35**, 2α, 3β-dihydroxyurs-12-en-28-oic acid **36**, 2α, 3β, 24-trihydroxyolean-12-en-28-oic acid **37** and 2α,3β,24-trihydroxyurs-12-en-28-oic acid **38**. 

Daucosterol **39**, ursolic acid **31** and 2α,3β,24-trihydroxy-12-en-28-ursolic acid **40** were obtained from the ethyl acetate fraction of *H. cuspidatus* aerial parts. The structures of these compounds were confirmed via analysis of mass and NMR data and compared with previously published data [77].

## 6. Phenolic Acids and Their Derivatives

**Figure 4:**
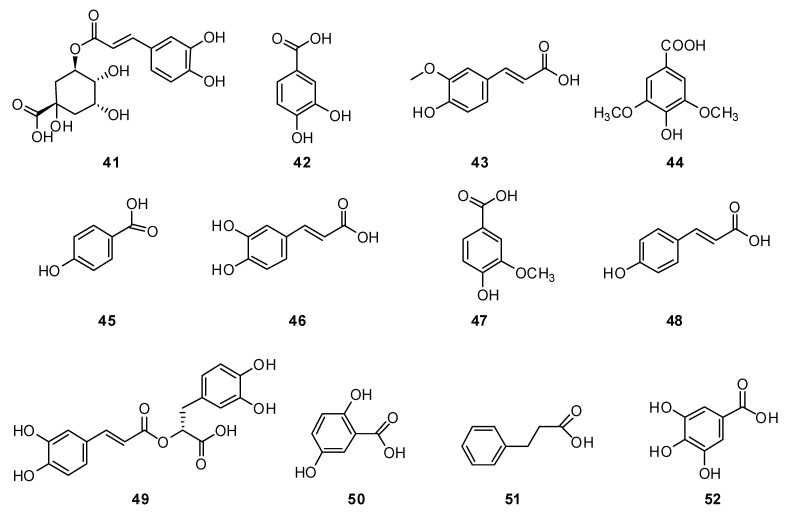
Chemical structures of phenolic acids from plants of the genus Hyssop.

To date, about thirty phenolic acids and their derivatives have been isolated from the genus *Hyssopus*, including chlorogenic acid **41**, protocatechuic acid **41**, ferulic acid **43**, lilac acid **44**, hydroxybenzoic acid **45**, caffeic acid **46**, vanillic acid **47**, p-coumaric acid **48**, rosemary acid **49**, gentisic acid **50** and phenylpropane **51** (Figure 4). These remain characteristic of the genus and are usually present in most *Hyssopus* species [78,79,80,81,82].

In terms of the dry raw materials, HPTLC analysis showed the presence of caffeic acid **46** (0.0064%) and ferulic acid **43** (0.034%) in the extract of *H. officinalis* [83].

HPLC DAD identified the following three phenolic acids: caffeic **46** (RT 8.65 min), ferulic **43** (RT 15.55 min) and rosemary **49** (RT 22.81 min) in a 70% ethanol extract of *H. cuspidatus* collected in 2018–2019. The contents of caffeic **46**, ferulic **43**, and rosmarinic acids **49** were 0.04–0.06%, 0.01–0.08 and 0.12–0.13% [84].

*H. officinalis* stem extract demonstrated the highest amount of total phenolic content at 374.60 ± 15.7 mg/g of gallic acid **52** [85].

**Figure 5:**
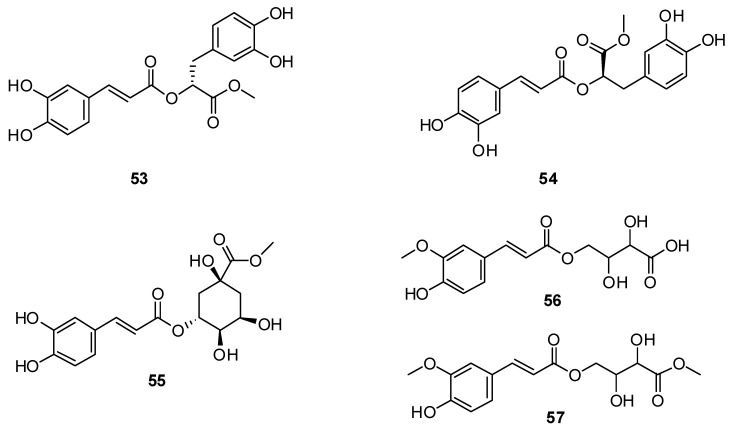
Chemical structures of derivatives of phenolic compounds from plants of the genus Hyssop.

From 17 kg of *H. cuspidatus*, 17 compounds were obtained, from which rosmarinic acid **49** and methyl rosmarinate **53**, 4-O-caffeoylquinic acid methyl ester **54**, 3-O-caffeoylquinic acid methyl ester **55** and caffeic acid **46** were isolated for the first time [77]. In addition, two new phenolic acids (Figure 5), (E)-2,3-dihydroxy-4-((3-(4-hydroxy-3-methoxyphenyl)acryloyl)oxy)butanoic acid **56** and (E)-methyl 2,3-dihydroxy-4-((3-(4-hydroxy-3-methoxyphenyl)acryloyl)oxy)butanoate **57**, were identified for the first time from *H. cuspidatus*, along with eleven known polyphenolic compounds [86].

Hydroxycinnamic acids were revealed as composing the majority of the extract isolated from *H. officinalis*, among which rosmarinic acid dominates **49**. The content of vitamins was also determined in this extract including levels of ascorbic acid (9.50 mg/100 g) and carotenoids (0.66 mg/100 g) [67].

**Figure 6:**
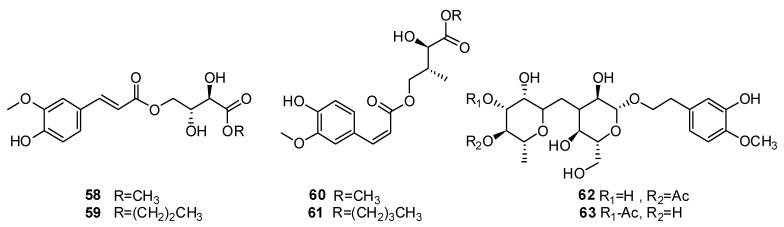
Chemical structures of new compounds from *H. cuspidatus*.

*H. cuspidatus* is a famous spice in Central Asia. In addition to the essential oil, non-volatile new compounds have been isolated from this plant species. The authors of [87] identified 64 compounds using LC-MS/MS, with phenolic compounds being the dominant components. The systematic separation and purification of *H. cuspidatus* ethanol extract resulted in the isolation of 34 compounds. The following 6 compounds (Figure 6) were identified as new compounds: hyssopusine A **58**, hyssopusine B **59**, hyssopusine C **60**, hyssopusine D **61**, 4′ ′-acetyldarendoside A **62** and 3′ ′-acetyldarendoside A—**63**, and 18 compounds were isolated from *H. cuspidatus* extract for the first time. 

**Figure 7:**
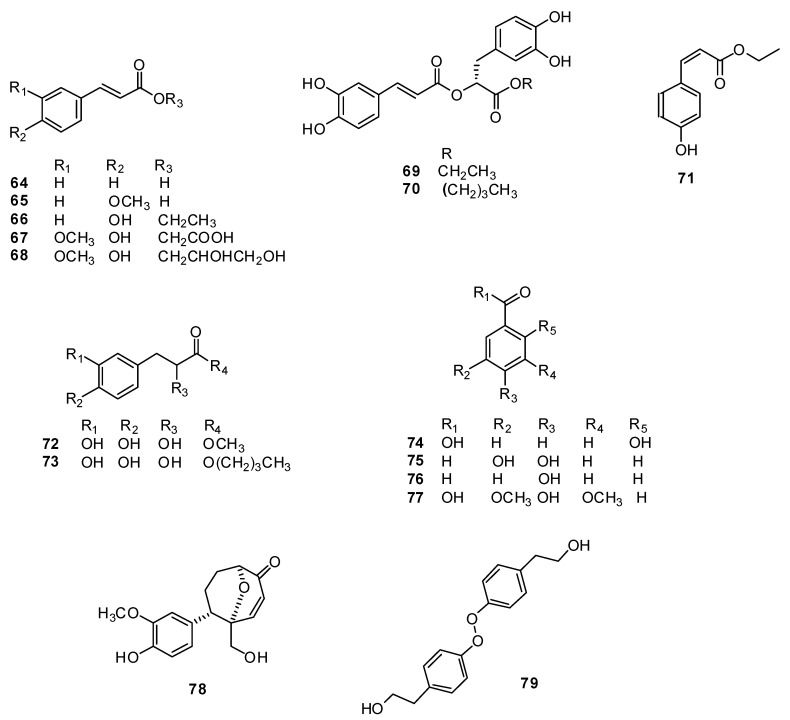
Chemical structures of molecules of polyphenolic compounds from plants of the genus Hyssop.

Among them were the following: *€*-cinnamic acid **64**, 4-methoxycinnamic acid **65**, ethyl *p*-coumarate **66**, ferulic acid **43**, carboxymethyl isoferulate **67**, 1-O-feruloylglycerol **68**, rosmarinic acid **49**, methyl rosmarinate **53**, ethyl rosmarinate **69**, butyl rosmarinate **70**, cis-p-coumaric acid ethyl ester **71**, 5R-5-hydroxy methyl-2 (5H)-furanone **72**, n-butyl-3,4-dihydroxy-phenyllactate **73**, p-hydroxybenzoic acid **45**, salicylic acid **74**, protocatechuic aldehyde **75**, p-hydroxybenzaldehyde **76**, syringic acid **77**, (+)-ligusticumtone **78** and p-hydroxy phenethyl alcohol **79** (Figure 7).

## 7. Compounds of a Flavonoid and Flavone Glycoside Nature

At present, about thirty flavonoids (flavones, flavanones, flavonols and flavanols) and their derivatives have been identified, isolated from various species of *Hyssopus*.

**Figure 8:**
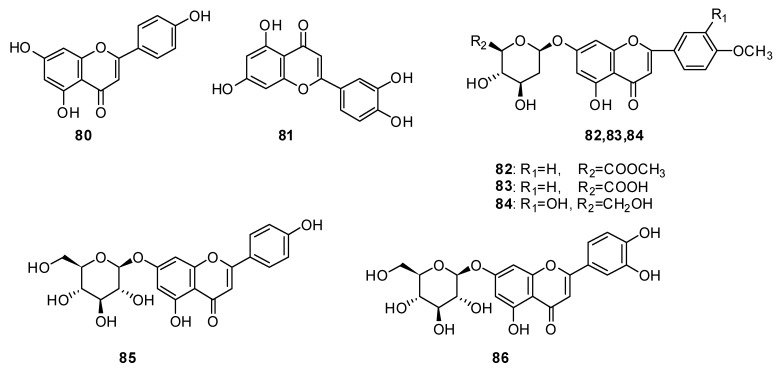
Chemical structures of isolated flavonoids and flavonoid glycosides from plants of the genus Hyssop. Part 1.

Apigenin **80**, luteolin **81**, acacetin-7-O-β−methyl glucuronide **82**, acacetin-7-O-β−glucuronide **83**, diosmetin 7-O-β−D-glucoside **84**, apigenin 7-O-β−glucoside **85**, luteolin-7-O-β-D-glucoside **86** and luteolin-7-O-β−D-galactoside **87** were first isolated from the genus *H. officinalis* (Figure 8) [88].

**Figure 9:**
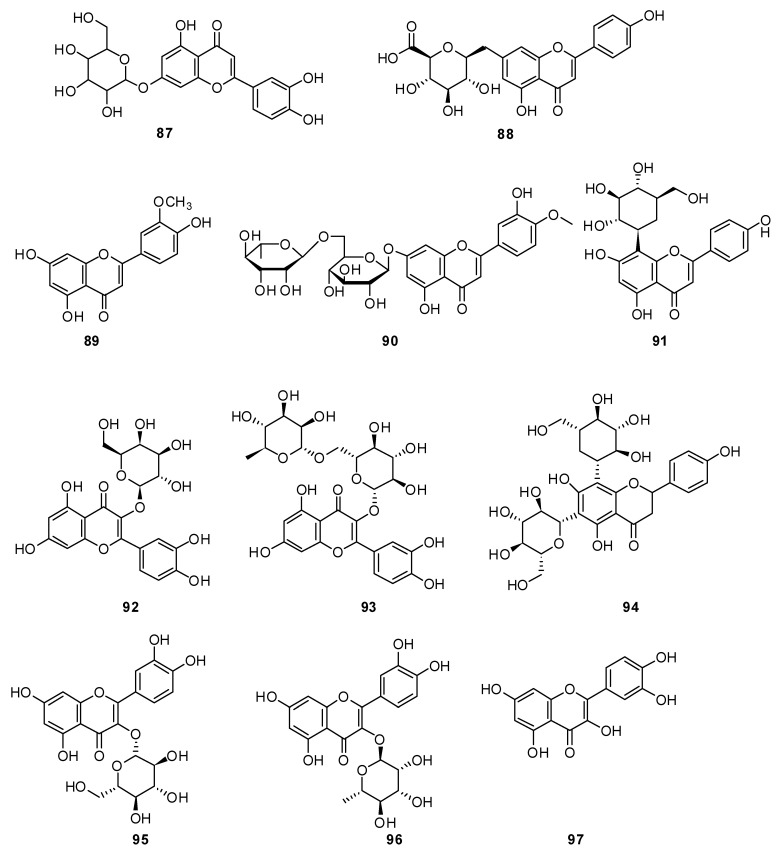
Chemical structures of isolated flavonoids and flavonoid glycosides from plants of the genus Hyssop. Part 2.

Apigenin 7-O-β-D-glucuronide **88** was determined to be the main flavonoid of *H. officinalis* from Iran [48]. The following six flavonoids were isolated individually from the herb *H. officinalis*: chrysoeriol **89**, diosmin **90**, vitexin **91**, hyperoside **92**, rutin **93** and vicenin **94** (Figure 9) [89]. 

Three flavonoid glycosides were detected in *H. officinalis* using HPLC-MS—isoquercitrin **95**, rutin **93** and quercitrin **96**, as well as two flavonoid aglycones—quercetin **97** and luteolin **81**. Isoquercitrin **95** was the flavonoid discovered in the highest amount (32.78 ± 0.23 µg/g) [90].

**Figure 10:**
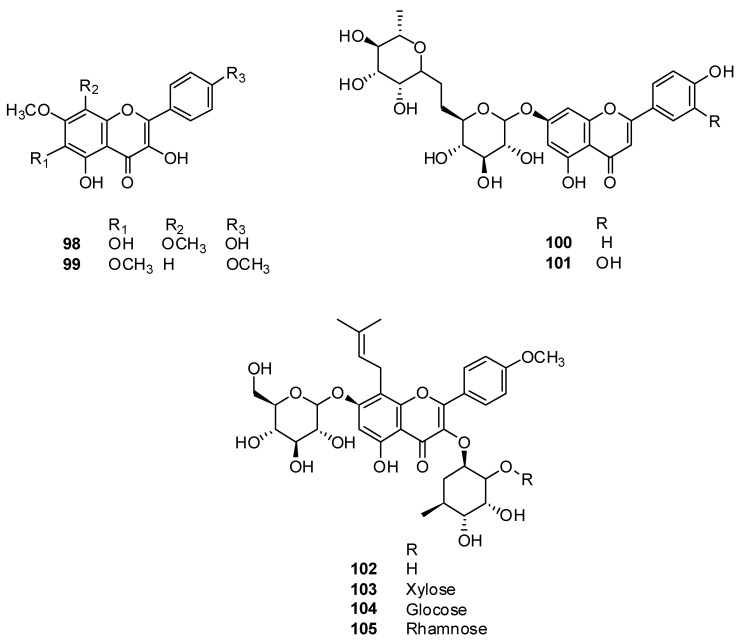
Chemical structures of isolated flavonoids and flavnoid glycosides from *H. cuspidatus*.

In China, scientists isolated for the first time eight flavonoids (Figure 10) from the *H. cuspidatus* herb: 5,6,4′-trihydroxyl-7,8-dimethoxyflavone **98**, salvigenin **99**, apigenin-7-rutinoside **100**, luteolin-7-O-α-L-rhamnosyl (1 → 6)-β-D-glucoside **101**, icariin **102**, 4-methoxy-5-hydroxy-8–3,3-dimethylallyl flavone-3-O-β-D-xylopyranosyl (1 → 2)-α-L-rhamnopyranoside-7-O-β-D-glucopyranoside **103**, 4′-methoxy-5-hydroxy-8–3,3-dimethylallyl flavone-3-O-β-D-glucopyranosyl (1 → 2)-α-L-rhamnopyranoside-7-O-β-D-glucopyranoside **104** and 4′-methoxy-5-hydroxy-8–3,3-dimethylallylflavone-3-O-β-L-rhamnopyranosyl (1 → 2)-α-L-rhamnopyranoside-7-O-β-D-glucopyranoside **105** [87]. 

## 8. Other Connections

**Figure 11:**
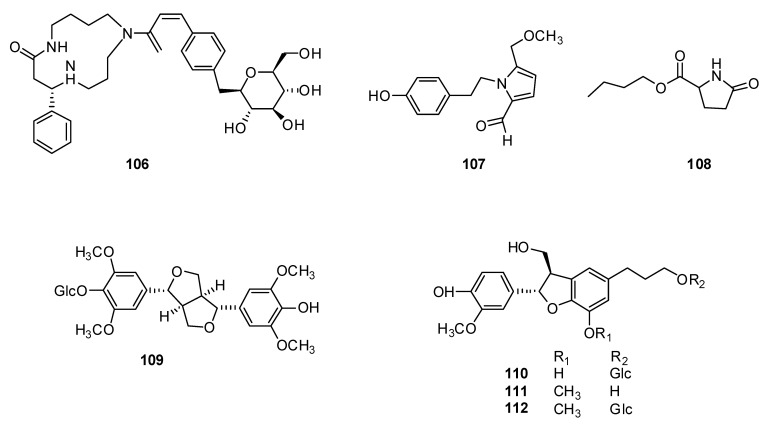
Chemical structures of isolated nitrogen-containing and polyphenolic compounds from *H. cuspidatus*.

In addition, Chinese scientists isolated a new macrocyclic spermidine alkaloid, hyssopusizine **106**, from an *H. cuspidatus* 95% ethanol extract [91] with 16 known compounds; this included for the first time the nitrogen-containing compounds (Figure 11) pyrrolezanthine-6-methylether **107** and n-butyl pyroglutamate **108** from the plants of the genus *Hyssopus*.

In addition, the lignan-type compounds syringaresinol 4′-O-β-D-glucopyranoside **109**, dihydrodehydrodiconiferyl alcohol 9′-O-β-D-glucopyranoside **110**, dihydrodehydrodiconiferyl alcohol **111** and (7R, 8S)-4,3′,9-trihydroxyl-3-methoxyl-7,8-dihydro-benzofuran-1′-propyl-neolignan 9′-O-β-D-glucopyranoside **112** were isolated from this compound [91].

**Figure 12:**
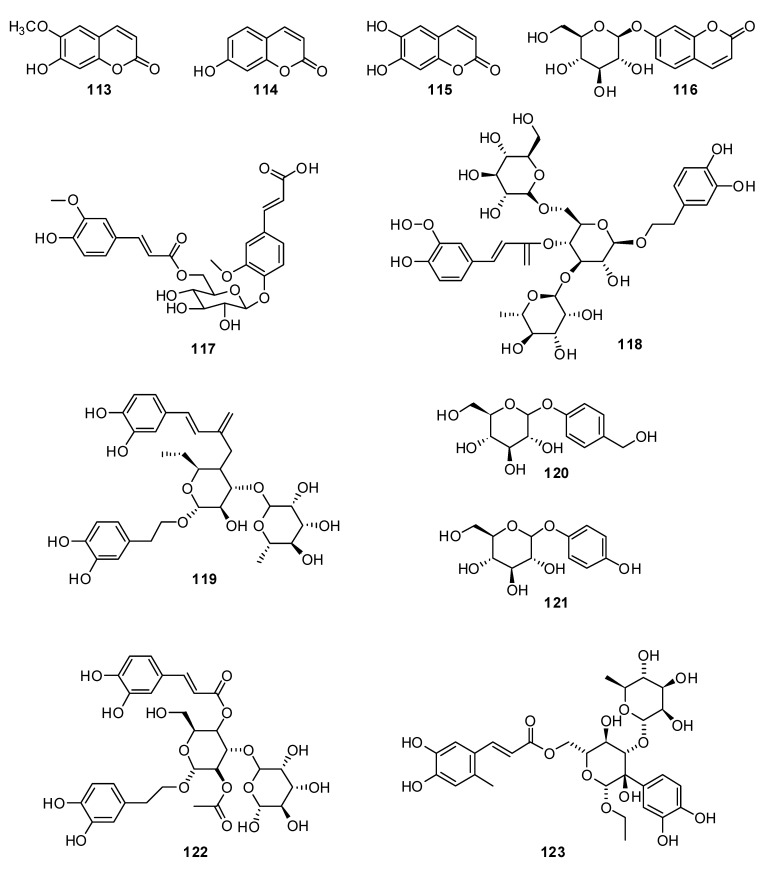
Chemical structures of isolated polyphenolic compounds from *H. cuspidatus*.

When conducting a qualitative analysis of the various groups of natural compounds in the *H. officinalis* herb, it is to be expected that phenolic compounds (coumarins, flavonoids, phenolcarboxylic acids, tannins, mainly condensed groups), polysaccharides, nitrogen-containing compounds (amino acids, nitrogenous bases), organic acids (citric, oxalic acid, tartaric acid, ascorbic acid), triterpene compounds and carotenoids will be encountered [89]. When quantitatively determined, it was found that the content of the sum of the nitrogenous bases in the studied *H. officinalis* herb ranged from 0.50% to 0.57% (including choline—from 0.08% to 0.10%; ascorbic acid—from 0.13% to 0.38%; the amount of free organic acids—from 5.07% to 13.87%; triterpene compounds—from 0.04% to 0.08%; tannins—from 18.32% to 19.24%; carotenoids—5.70 mg/g and essential oil—from 0.60% to 1.98%). The amino acids of the *H. officinalis* herb were represented by the following 11 compounds: aspartic acid, threonine, serine, glycine, alanine, valine, leucine, tyrosine, lysine, phenylalanine and histidine. The dominant free amino acids were threonine and serine (7.63 mg/g). The raw materials of *H. officinalis* were established to contain inorganic substances such as iron, potassium, sodium, calcium, magnesium, aluminum, silicon, copper, zinc, silver, strontium, phosphorus, manganese and titanium. The predominant macroelements were potassium (1%), calcium (1%) and magnesium (1%), and the microelements were silicon (0.3%) and aluminum (0.1%). Two coumarins (Figure 12), scopoletin **113** and umbelliferon **114**, were also isolated from a 70% ethanol extract.

The coumarins esculetin **114** and umbelliferon hexoside **116** were isolated and identified in *H. cuspidatus* [91].

The new glycoside hyssopuside **117** was isolated together with other phenolic glycosides **118–123** from *H. cuspidatus* [83].

## 9. Biological Activities

The species within the genus *Hyssopus* have primarily been evaluated for their potential anti-inflammatory, antioxidant, antibacterial, antifungal, and anti-asthmatic effects [47,52,53,64].

At present, *H. officinalis* is widely used in the culinary, food and medical industries. The green shoots of hyssop, cut before flowering, are used for medicinal purposes. In folk medicine, hyssop is used in the form of an infusion as an expectorant for chronic bronchitis, for asthma, as well as in chronic gastritis, as a wound-healing agent and an anti-sweating agent. A decoction of hyssop is used to wash the eyes and mouth during inflammatory processes and is used as a means of improving digestion [79].

An infusion of hyssop is recommended for older people as a general health drink and is also used for compresses for rheumatism, bruises, conjunctivitis and as a weak diuretic and carminative.

Using hyssop greens in the diet promotes digestion, increases appetite, tones the body and acts as a general tonic. Hyssop raw materials are used for bronchitis, catarrh of the upper respiratory tract, bronchial asthma, angina pectoris, neuroses, joint diseases, chronic colitis, flatulence, diabetes, as an anthelmintic and also as an antiseptic. Infusions and decoctions are used externally to wash the eyes, for stomatitis, diseases of the nasopharynx, for compresses for hemorrhages, bruises and as a wound-healing agent [83].

A range of the main pharmacological effects of different *Hyssopus* species are presented in Table 3.

## 10. Conclusions

The chemical composition of the plant *H. officinalis*, which is one of the most popular species, distributed mainly from the Eastern Mediterranean to Central Asia, has been sufficiently studied. The plant has traditionally been used for medicinal purposes. The raw materials contain essential oils, flavonoids and polyphenolic acids. The flower tips contain ursolic acid and the glucoside diosmin. The main components of the essential oil are bicyclic monoterpenes (L-pinocamphene, cis-pinocamphone, pinocarvone, β-pinene), depending on the chemotype of the plant. The main components of *H. officinalis* include apigenin, quercetin, diosmin, luteolin and their glycosides, chlorogenic, protocatechuic, ferulic, lilac, p-hydroxybenzoic, caffeic and other acids. *H. officinalis* has a moderate antioxidant effect and antimicrobial activity against Gram-positive and Gram-negative bacteria; pronounced antifungal, insecticidal and antiviral properties were also identified in vitro. The studies on animal models have indicated muscle relaxant and antiplatelet properties. However, human studies, investigation of adverse reactions and clinical trials are lacking and further study is needed.

For the *H. ambiguous* plant growing in Central Kazakhstan, the chemical composition of the essential oil was ascertained. The main component is 1,8-cineol; therefore, its antimicrobial properties were studied and, based on the data obtained, methods were developed for obtaining essential oil compositions with a pleasant odor for the further development of a high-quality inhalation form. In addition, the creation of relatively inexpensive therapeutic, treatment and prophylactic agents that can effectively combat the infectious diseases of the upper respiratory tract was investigated. The plant was not studied for the content of other biologically active compounds.

*H. cuspidatus* is a famous Chinese plant and is frequently used in traditional Uyghur folk medicine to treat cough, asthma, bronchitis and rheumatism. The raw material contains essential oil, which is currently the most studied functional natural component; additionally, polyphenols, flavonoids, triterpenes and steroids are studied as the other principal components. Currently, a number of new compounds have been isolated from this plant species. Modern pharmacological research has shown that *H. cuspidatus* can reduce or improve airway inflammation, lower blood sugar, eliminate phlegm and relieve cough, and also has many biological properties such as antibacterial, antioxidant and antitumor.

*H. seravschanicus* is distributed in the mountain forests, valleys and gorges of Central Asia. The plant has been used in medicinal practice since ancient times; however, in modern folk and scientific medicine, it is only occasionally used. The plant is cultivated in some European countries. The chemical composition of the plant has not been sufficiently studied, but it has been identified that it contains essential oil, flavonoids, glycosides and steroids. In Tajikistan, the plant is being used to develop antimicrobial medicinal forms based on the essential oil.

The scientific research has also confirmed its antispasmodic properties and has shown that hyssop essential oil has both an antiseptic and sedative effect.

The long-term studies from scientific centers in a number of countries, including China, Russia, Iran, Bulgaria, Turkey, Bulgaria, Tajikistan, Kazakhstan, etc., have focused on the chemical composition and pharmacological activity of the *Hyssopus* species and have shown that the chemical composition of the plants varies significantly, based on abiotic and biotic factors, as well as extraction methods. The variability in the chemical composition (both qualitative and quantitative) of a plant extract or essential oil can lead to significant differences in its pharmacological activity. Currently, not all *Hyssopus* species have been studied for their chemical composition and biological activity.

At the same time, there is no information about existing or proposed non-clinical and clinical developments and side effects, which therefore requires additional research.

The extracts, essential oils and individual compounds isolated from *Hyssopus* are attracting increasing attention as a valuable source for drug development and complementary health products. At the same time, it is necessary to take into account the isolation procedures, different chemotypes, time and place of collection and different biological activities for the development of new drugs based on the *Hyssopus* species; it is clear that plants of the genus require more careful and in-depth study. 

Thus, the biologically active compounds of plants of the genus *Hyssopus* are a promising source for the development and introduction into medicine of new innovative highly effective herbal medicines with a wide spectrum of action.

## Figures and Tables

**Table 1:** The morphological properties of species of the genus *Hyssopus* growing in Kazakhstan.

Indicators	*H. cuspidatus*	*H. ambiguus*	*H. macranthus*	*H. seravschanicus*	*H. latilabiatus*	*H. officinalis*	*H. subulifolius*
Life form	Subshrub	Subshrub	Subshrub	Subshrub	Subshrub	Subshrub	Subshrub
Leaves	Narrowly linear, with non-folding edges, with an awl-shaped tip at the apex	Glabrous, entire, narrowly linear, with edges turning downwards, with a vein protruding from the underside	Sessile, lily, pointed, narrowed at the base, twisting	Leaves linear, almost glabrous, with sparse short hairs, sharp, with curled edges	Leaves sessile, linear, glandular, base wedge-shaped, edges curving inward	Leaves are lanceolate, short-petiolate, short-hairy	Leaves are small, needle-shaped, short-petiolate, glabrous
Inflorescence	Multi-flowered, thin, tapering towards the apex	Multi-flowered, unbranched, dense	Thin, multi-flowered, tapering towards the apex	Long, narrow, consists of loose whorls	Short, apical, spicate, few flowers	Long, spicate, flowers sit in the axils of the upper leaves	Long, spicate, covered with small white hairs
Calyx	Regular, with 5 awl-shaped pointed teeth	4–6 mm long, with five almost identical teeth, equal to one-third of the total length of the calyx	4–6 mm long, covered with short hairs along the veins and along the edge of triangular, sharp teeth	5–6 mm long, with triangular sharp teeth, two times shorter than the tube, painted blue	Tubular-bell-shaped, 5–6 mm long, purple, with five teeth, pubescent, glandular at the apex	5–6 mm long, with five teeth, increased to one-third of the total length of calyx, with short hairs along veins, purple color	6–8 mm long, purple, with five triangular teeth, pubescent with hairs along the veins
Corolla	Blue, up to 12 mm long, with a short tube, two-lipped, upper lip two-lobed, shorter than the lower, lower three-lobed, with a large middle lobe	Bluish–blue, 0.8–1 cm long, two-lipped, the upper lip is flat, bilobed, the lower lip is three-lobed with a large middle lobe	10–15 mm long, blue–violet, short-pubescent on the outside, two-lipped, upper lip slightly notched, smaller than the lower, three-lobed. On the lower lip, the middle lobe is two times wider than the lateral ones	Blue–violet, about 1 cm long, with a narrow tube, about 5 mm, the upper lip is ovoid, equal to the lower, the middle large lobe is strongly prominent on the lower lip	Purple, 12–13 mm long, pubescent, glandular; the upper lip is straight, oblong; lower lip is broadened; middle lobe up to 1 mm, considerably wider than lateral lobes; lateral lobes are ovate	Purple, 10–15 mm long, two-lipped, upper lip with notch, shorter than lower lip. The lower lip with well-defined, downwardly bent middle lobe	White, two-lipped, 12–16 mm long, the upper lip with notch, short; the lower lip is three-lobed, large; middle lobe is round
Habitat	Grows in feather-fescue sepia, on rocky mountain slopes and on pebbles	Grows on crushed and rocky mountain slopes, on pebbles	Grows on saline flood meadows, rocky and gravelly slopes of hills and hills, on pebbles and coarse sandy soils	Grows on rocky and gravelly slopes and trails of mountains, on steppe areas	Grows on dry and rocky slopes, on stony screes	Grows in steppes, on dry hills, rocky slopes of hillsides	Grows on dry and stony soils, in dry forests and shrub thickets
Distribution in the Republic of Kazakhstan	Altai, Tarbagatai, Dzungarian Alatau	Irtysh, Eastern Small Hills, Karkaralinsky, Altai, Tarbagatai, Dzhungar Alatau	Irtysh, Western and Eastern small hills, Karkaraly, Zaisan, Bal-khash-Alakol, Altai, Tarbagatai	Kyrgyz Alatau, Western Tien Shan	Absent	Absent	Absent
General distribution *	Altai, Kazakhstan, Mongolia, China (Xinjiang)	Altai, Kazakhstan, Mongolia, West Siberia	Endemic of Kazakhstan	Afghanistan, Kirgizstan, Pakistan, Tadzhikistan, Uzbekistan	Endemic of China (Xinjiang)	South and central Europe, South Siberia, Mediterranean region, North Caucasus, Turkey, North Africa (Morocco)	Endemic of Afghanistan

* Distribution of natural *Hyssopus* species in the world (according to POWO [3]). Based on international theories, the following four species of this genus grow in Kazakhstan: *H. cuspidatus*, *H. ambiguus*, *H. macranthus* and *H. seravschanicus*. All species in terms of life forms are subshrubs, with simple sessile leaves and multi-flowered inflorescences.

**Table 2:** Chemical composition of essential oils of different species in the genus *Hyssopus* L.

Species	Location	Number of Identified Compounds	Main Compounds	Reference
*H. officinalis*, culture	Poland	From 27 to 36	Cis-pinocamphone (40.07–45.45%)	[12]
*H. ambiguus*	Kazakhstan	9	1,8-cineole (36.0–43.5%)	[24,25]
*H. cuspidatus*	Kazakhstan	83	Pinocarvone (27.06%), 1,8-cineole (10.76%), cis-pinocarveol (9.57%)	[26]
*H. cuspidatus*	China	38	Verbenone (23.84%), β-pinene (19.76%), pinocamphone (17.95%), 1,8-cineole (7.16%), myrtenol (7.06%)	[27]
*H. cuspidatus*	China	36	Germacrene D (18.67%), hexadecanoic acid (17.53%), germacrene B (15.61%), trans-caryophyllene (8.04%)	[28]
*H. cuspidatus*	China	39	Thymol (19.65%), pinocamphone (15.30%), γ-terpinene (14.63%), *p*-cymene (7.49%), β-pinene (6.57%)	[29]
*H. officinalis*	Iran	14	Camphor (23.61%), β-pinene (21.91%)	[30]
*H. officinalis*	Cultivated in Serbia	18	cis-pinocamphone (42.9%), pinocamphone (14.1%), germacrene-D-11-ol (5.7%), elemol (5.6%)	[31]
*H. officinalis*	Egypt	26	Cis-pinocamphone (34.00%), pinocamphone (21.27%), β-pinene (13.19%), β-phellandrene (13.10)	[32]
*H. officinalis*	Turkey	34	Cis-pinocamphone (57.27%), β-pinene (7.23%), terpinen-4-ol (7.13%), pinocarvone (6.49%)	[33]
*H. officinalis* subsp. *angustifolius*	Turkey	51	Pinocarvone (27.1%), β-pinene (19.0%), cis-pinocamphone (13.6%)	[34]
*H. officinalis* subsp. *officinalis* L.	Serbia	59	Cis-pinocamphone in f. albus (16.4%), in f. cyaneus (22.3%), in f. ruber (58.3%)	[35]
*H. officinalis*	East Lithuania	63	Pinocarvone (21.1–28.1%), cis-pinocamphone (11.5–15.9%), β-pinene (7.0–11.4%), germacrene D (3.7–5.5%), hedycaryol (4.1–4.8%) in four oils, cis-pinocamphone (16.8–33.6%) in two oils	[36]
*H. officinalis*	Russia	From 31 to 37	White-flowered pinocamphone up to 44.99%, blue-flowered pinocamphone up to 20.85%, pink-flowered pinocamphone up to 45.23%	[37]
*H. officinalis*	Poland	5	Cis-pinocamphone (33.52%), pinocamphone (28.67%), β-pinene (8.12%), elemol (5.86%)	[38]
*H. officinalis*	Iran	17	pinocamphone (53.93%)	[39]
*H. officinalis*	Russia	27	Pinocamphone (63.55%)	[40]
*H. officinalis* f. cyaneus	Russia, cultivated	68	Pinocamphone (70%)	[41]
*H. officinalis* L. subsp. *angustifolius*	Iran	25 and 22	Purple landrace was cis-pinocamphone (55.14%), β-pinene (17.06%), pinocamphone (3.50%); White landrace of hyssop camphor (31.85%), cis-pinocamphone (30.11%), β-pinene (12.26%), pinocamphone (6.09%)	[42]
*H. officinalis* ssp. *officinalis*	India	21	Pinocamphone (49.1%), β-pinene (18.4%), cis-pinocamphone (9.7%)	[43]
*H. officinalis*	Turkey	24	Pinocarvone (29.2%), trans-pinocamphone (27.2%), β-pinene (17.6%), cis-camphone (4.7%)	[44]
*H. officinalis*	Egypt	33	Cis-pinocamphone (26.85%), β-pinene (20.43%), pinocamphone (15.97%), α-elemol (7.96%)	[45]
*H. officinalis*	India	33	cis-pinocamphone (53.34%), β-pinene (9.91%), limonene (7.19%)	[46]
*H. officinalis*	Serbia	74	Pinocamphone (41.2%)	[47]
*H. officinalis*	Iran	19	Myrtenyl acetate (74.08%), camphor (6.76%), germacrene (3.39%)	[48]
*H. officinalis*	Poland	52	Cis-pinocamphone (22.53–28.74%), pinocamphone (11.41–17.99%), β-pinene (6.69–12.01%), elemol (5.02–7.57%), germacrene D (3.14–6.98%)	[49]
*H. officinalis*	Spain	44	1,8-cineole (53%), β-pinene (16%)	[50]
*H. officinalis*	Poland	74	Cis-pinocamphone (20.05–43.02%), pinocamphone (1.68–19.62%)	[51]
*H. officinalis*	Poland	50	White-flowered pinocamphone (51%), pink-flowered pinocamphone (28.8%), cis-pinocamphone (21.9%)	[52]
*H. officinalis*	Bulgaria	46	Cis-pinocamphone (48.98–50.77%), β-pinene (13.38–13.54%), pinocamphone (5.78–5.94%)	[53]
*H. officinalis*	Iran	36	Cis-pinocamphone (38.47%), pinocamphone (13.32%), pinocarvone (5.34%)	[54]
*H. officinalis*	Cultivated in Bulgaria	55	Cis-pinocamphone (40.2%), pinocamphone (10.3%), β-pinene (14.2%)	[55]
*H. officinalis*	Egypt	-	White-flowered β-pinene (19.60%), pinocamphone (19.20%), camphor (16.3%)	[56]
*H. officinalis*	India	47	Cis-pinocamphone (38.1%), pinocarvone (20.3%), 1,8-cineole (12.2%)	[57]
*H. officinalis* var. *decumbens*	France	16	Linalool (49.6%), 1,8-cineole (13.3%), limonene (5.4%)	[58]
*H. officinalis*	Montenegro	45	Methyl eugenol (38.3%), limonene (37.4%), β-pinene (9.6%)	[59]
*H. officinalis*	Yugoslavia		Cis-pinocamphone (46.1%)	[60]
*H. officinalis*	Spain	21	1,8-cineole (52.89%), β-pinene (16.82%)	[61]
*H. officinalis* L. subsp. *angustifolius* (Bieb.)	Turkey	34	Pinocarvone (36.3%), pinocamphone (19.6%), β-pinene (10.6%), 1,8-cineole (7.2%), cis-pinocamphone (5.3%)	[62]
*H. seravschanicus*	Ukraine, in culture	27	Cis-pinocamphone (61.58%)	[63]
*H. seravschanicus*	Tajikistan	87	Cis-pinocamphone (57.0–88.9%), β-pinene (0.4–6.0%), 1,8-cineole (1.8–3.6%), camphor (0.5–4.0%), spathulenol (0.1–5.0%)	[64]
*H. cretaceus*	Russia	45	Cis-pinocamphone (60%), pinene (12.78%), myrtenyl acetate (7.17%)	[65]
*H. officinalis*	Russia, Crym	58	Cis-pinocamphone (29.7–58.4%), pinocamphone (15.2–23.3%)	[66]

**Table 3:** Pharmacological activity of extracts, essential oils and individual compounds isolated from plants of the species *Hyssopus* L.

Pharmacological Activity	Test Sample	Place of Growth	References
Antioxidant activity	*H. officinalis* essential oil; IC_50_ = 24.0 ± 0.2 µg/mL	Serbia	[47]
	*H. officinalis* methanolic extract; IC_50_ = 0.50 µg/mL	India	[83]
	*H. cuspidatus* 70% ethanol extract AOA; IC_50_ of 0.0245 mg/mL	China	[84]
	*H. cuspidatus* compounds **109**–**111** ABTS; IC_50_ 27.2–45.5 μM	China	[91]
	*H. cuspidatus* compounds **48**, **52**, **69** showed high AOA	China	[87]
	*H. officinalis* methanolic extracts DPPH; IC_50_ = 56.04–199.89 µg/mL, FRAP = 0.667–0.959 mmol Fe^2+/^g	Serbia	[92]
	N-butanol extract; IC_50_ = 25 mg/mL	Iran	[90]
	*H. officinalis* extract had a moderate lipid peroxidation and antioxidant activity	Iran	[93]
Antimicrobial activity	*H. officinalis* essential oil MIC: *B. cereus*, 14.20 µL/mL; *E. coli*, 227.25 µL/mL; *E. faecalis*, 454.50 µL/mL; *P. aeruginosa*, 454.50 µL/mL; *S. enteritidis*, 227.25 µL/mL; *S. aureus*, 227.25 µL/mL; *S. epidermidis*, 227.25 µL/mL; *P. hauseri*, 227.25 µL/mL *H. officinalis* essential oil MBC: *B. cereus*, 28.40 µL/mL, 227.25 µL/mL; *E. faecalis*, 454.50 µL/mL; *P. aeruginosa*, 454.50 µL/mL; *S. enteritidis*, 227.25 µL/mL; *S. aureus*, 227.25 µL/mL; *S. epidermidis*, 227.25 µL/mL; *P. hauseri*, 454.50 µL/mL	Serbia	[51]
	*H. officinalis* essential oil growth of inhibition zones in the case of typical strains: *S. aureus*, 17.00 ± 0.20 mm; *E. coli*, 14.00 ± 0.56 mm; *E. faecalis*, 8.33 ± 0.33 mm; *S. pyogenes*, 11.00 ± 0.57 mm; *C. albicans*, 11.50 ± 0.20 mm *H. officinalis* essential oil growth of inhibition zones in the case of clinical strains: *S. aureus*, 20.00 ± 0.10 mm; *E. coli*, 10.66 ± 0.88 mm; *S. pyogenes*, 11.33 ± 0.33 mm; *C. albicans*, 12.00 ± 0.80 mm	Czech Republic	[94]
	*H. officinalis* L. subsp. *aristatus* (Godr.) Nyman essential oil MIC against *S. aureus* and *E. coli*, 400 µg/mL	Montenegro and Serbia	[95]
	*H. officinalis* ethanolic extract biofilm formation against *E. coli* (95%). *K. pneumoniae* biofilm had a resistant biofilm structure between all tested bacteria (16.41%)	Iran	[96]
	*H. officinalis* ethanolic extract MIC: *B. cereus*, 1.562 µg/µL; *S. marcescens*, 6.25 µg/µL; *P. aeruginosa*, 3.125 µg/µL	Iran	[93]
	*H. officinalis* hydrolate had activity against natural test objects and recombinant bacteria *E. coli* (p Xen-lux)	Russia	[7]
	*H. seravschanicus* essential oil MIC: *B. cereus* and *S. aureus*, 312 µg/mL; *P. aeruginosa*, *E. coli*, *C. albicans* and *A. niger*, 625 µg/mL	Tajikistan	[64]
	*H. officinalis* methanolic extract MIC: *B. cereus* and *S. aureus*, 25 mg/mL; *P. aeruginosa* and *E. coli*, 50 mg/mL *H. officinalis* methanolic extract MBC: *B. cereus* and *S. aureus*, 50 mg/mL; *P. aeruginosa* and *E. coli*, 100 mg/mL	Iran	[97]
	*H. officinalis* L. white-flowered essential oil MIC and MBC: *S. aureus* (10 mg/mL, 20 mg/mL), *S. epidermidis* (5 mg/mL, 10 mg/mL), *B. subtilis* (5 mg/mL, 5 mg/mL), *M. luteus* (2.5 mg/mL, 5 mg/mL), *E. coli* (5 mg/mL, 10 mg/mL), *K. pneumoniae* (5 mg/mL, 10 mg/mL), *P. aeruginosa* (5 mg/mL, 10 mg/mL) *H. officinalis* L. pink-flowered essential oil MIC and MBC: *S. aureus* (5 mg/mL, 10 mg/mL), *S. epidermidis* (2.5 mg/mL, 5 mg/mL), *B. subtilis* (0.625 mg/mL, 2.5 mg/mL), *M. luteus* (2.5 mg/mL, 5 mg/mL), *E. coli* (5 mg/mL, 5 mg/mL), *K. pneumoniae* (5 mg/mL, 10 mg/mL), *P. aeruginosa* (5 mg/mL, 10 mg/mL)	Poland	[52]
Antifungal activity	Compounds **105**, **109**–**110** from *H. cuspidatus* exhibited inhibitory effects against the proliferation of *C. albicans* with inhibitory zone diameters from 7.5 to 12.0 mm	China	[91]
	*H. officinalis* essential oil showed activity against *S. pyogenes*, *S. aureus*, *C. albicans* and *E. coli* with inhibition zone diameters of 19.0 ± 0.1 mm, 18.0 ± 1.7 mm, 20.3 ± 1.8 mm and 15.0 ± 1.0 mm	Turkey	[33]
	*H. officinalis* L. var *decumbens* (Jordan & Fourr.) Briq. from France (Banon) and *H. officinalis* L. from Italy (Piedmont) essential oils were active against *C. albicans*, *C. krusei* and *C. tropicis*	France and Italy	[98]
	*H. officinalis* L. white-flowered essential oil MIC and MBC: *C. albicans* (0.625 mg/mL, 2.5 mg/mL), *C. parapsilosis* (1.25 mg/mL, 5 mg/mL) *H. officinalis* L. pink-flowered essential oil MIC and MBC: *C. albicans* (0.625 mg/mL, 2.5 mg/mL), *C. parapsilosis* (0.625 mg/mL, 1.25 mg/mL)	Poland	[52]
	*H. officinalis* essential oil demonstrated inhibition of ATPase enzyme and increased the membrane permeability in *Candida* species. This effect was caused due to synergistic effects of chemical constituents from essential oils like β-pinene, α-pinene, trans-pinocamphone and cis-pinocamphone	Bulgaria	[53]
Anti-inflammatory activity	Phenolic glycoside **116** isolated from *H. cuspidatus* could reduce NO production and inhibit TNF-α, IL-6 and IL-1β	China	[92]
	*H. cuspidatus* essential oil had an anti-inflammatory effect of 0.4 mL/kg, which exceeds aspirin. *H. cuspidatus* essential oil was noted in inhibiting the production of TNF-α, IL-1β, IL-6 and PGE2 and significantly reduced the MDA and NO levels	China	[99]
	*H. officinalis* extract at doses of 25, 50 and 75 mg/kg/bw (13.33 ± 3.1, 20 ± 3.1, 19.83 ± 2.8) demonstrated high anti-inflammatory effects against Xylene-induced ear edema	Iran	[100]
Anti-asthmatic activity	Treatment with *H. cuspidatus* extract reduced the amount of sputum and decreased the infiltration of inflammatory cells around the bronchi in mice. The extract had a significant ameliorative effect on ovalbumin-induced asthma	China	[101]
	*H. cuspidatus* ethanolic extract and the rosmarinic acid isolated from it had anti-asthmatic activity	China	[102]
	*H. officinalis* L. extract affected interleukin-4, -6 and -17 and interferon-γ levels in asthmatic mice and inhibited the invasion of EOS	China	[103]
Antitumor activity	*H. officinalis* L. essential oil using the MTT test showed antitumor activity against the tumor cell lines SW480, MDA-MB 231, HeLa and MRC-5	Serbia	[92]
	ZnO nanoparticles using *H. officinalis* extract disrupted spermatogenesis, the sperm maturation process and sperm motility. The IC_50_ for the PC3 cell line treated with ZnO nanoparticles for 24 and 48 h was recorded at 8.07 and 5 μg/mL, and induced apoptosis was 26.6% ± 0.05, 44% ± 0.12 and 80% ± 0.07 for the PC3 cells	Iran	[104]
	*H. officinalis* ethanolic extract concentration of 500 mg/mL showed 82% cytotoxic effect for breast cancer cells	India	[19]
Antidiabetic activity	Aerial parts of *H. officinalis* L. were screened for determination of antidiabetic activity using an alpha-amylase inhibition assay, namely, a starch iodine assay model, and found an IC_50_ = 0.8366 mg/mL	India	[105]
Antiviral activity	*H. officinalis* L. methanolic extract demonstrated antiviral effects against HSV at an oral dose of 125 mg/kg in mice	Iran	[106]
	*H. officinalis* methanolic extract demonstrated significant anti-HIV activity due to the high content of caffeic acid	USA	[107]
	The polysaccharide MAR-10 isolated from the methanol extract of *H. officinalis* leaves inhibited human immunodeficiency virus type 1 replication in HUT78 T cells and peripheral blood mononuclear cells in a concentration-dependent manner	China	[108]
Antispasmodic activity	*H. officinalis* L. essential oil inhibited the acetylcholine- and BaCl_2_-induced contractions, with an IC_50_ of 37 μg/mL and 60 μg/mL, respectively	China	[109]
Anti-leishmaniasis activity	*H. officinalis* extract ointment showed significant effectiveness against cutaneous leishmaniasis due to the release of nitric acid and tumor necrosis factor from the macrophages	Iran	[110]
Anticonvulsant activity	The water hyssop extracts, having a concentration of 100 mg/kg, showed anticonvulsant action and caused a significant increase in iNOS gene expression in the hippocampus	Iran	[111]
Insecticidal activity	*H. cuspidatus* essential oil possessed fumigant toxicity against *S. zeamais* adults, with an LD_50_ = 24.44 μg/adult and LC_50_ = 16.72 mg/L	China	[29]
Mosquito larvicidal activity	*H. officinalis* essential oil in an acute toxicity study against *Culex* mosquitos revealed an LC_50_ of more than 90 μL/L	France	[71]
Myorelaxation activity	The inhalation of hyssop essential oil increased the immobile position and may have caused a sedative effect in mice	Iran	[112]

## Data Availability

Not applicable.
